# A Demonstration of the Nerves of the Human Body; Consisting of Four Parts

**Published:** 1834-04-01

**Authors:** 


*A Demonstration of the Nerves of the Human Body; consisting of Four Parts.* By JOSEPH SWAN. Part IV. London. Folio.

THESE magnificent plates, containing the delineations of the spinal nerves, complete Mr. Swan's great work, and will add fresh lustre to his reputation.

As a dissector of the nerves, Mr. Swan has long been without an equal, and the previous parts of this unrivalled work have shown that our author has had the sense and spirit to employ artists worthy of embodying the results of his anatomical investigations. In a case of this kind, panegyric, however richly deserved, is superfluous; for every one will buy the book who can afford it. Had we room we should be tempted to make many quotations; for, though we must still leave behind the "animæ dimidium suæ,"—though we could not extract the drawings of West, nor the engravings of Finden,—yet the other half is so good, and Mr. Swan writes on the nerves with so masterly a command of the subject, that we would willingly linger over his ample pages, and teach our readers some of the more refined mysteries of dissection. As it is, we must terminate this brief notice with a single extract.

"Whoever prosecutes the anatomy of the nervous system attentively, adverting at the same time to its physiology and pathology, as far as these are already known, can hardly fail of being convinced that every organised part of the animal body is supplied with nerves; and, as different degrees of perception are called for in different parts, so these are more or less supplied with nervous branches accordingly. The organs of the senses are very important, and are therefore furnished with the greatest number. Next in importance are the muscles, whose actions are of such concern to the body; and these are furnished with nerves in proportion. The viscera, the glands, and blood-vessels, receive many nerves; but the bones, tendons, &c., are passive, and contain only a small proportion; no more indeed than might be supposed necessary for maintaining such a degree of living action as would preserve them from injury, and form that connexion with every other part required for the harmonious discharge of the functions of the whole. With such a variety in the distribution of nerves, can it be a matter of wonder, that there should be so much difficulty in tracing the small branches on the least sensible parts; or, because the demonstration cannot always be accomplished, are these to be considered destitute? Certainly not; for, if nerves have been satisfactorily exhibited under peculiarly favourable circumstances, the difficulty, or even the failure, of accomplishing such dissections generally, is no proof either against their reality in the few examples, or their existence in every subject.

"So complicated is the structure of an animal body, that however diligent the anatomist may be in acquiring the art of separating its different textures, it is impossible for him to see how the ultimate particles are disposed. The arteries, and veins, and absorbents, may be so filled with different substances, as to be traced with accuracy to the utmost degree of minuteness; but with the nerves it is very different; for these the anatomist can only trace to a certain extent with a sufficient assurance that what he is following is nerve. When he gets beyond this point, the surrounding parts become so similar to the nervous filaments as to make the separation and dissection difficult, but not so much so as has been generally imagined. By the most careful dissection nerves may be followed to their termination in a delicate membrane, which will be found, on investigation with a magnifying-glass, to consist of a flexus of minute filaments.

"Many are apt to be too much guided by notions they have early imbibed respecting the form, substance, &c. of various parts of the body, and cannot at first reconcile it to themselves with regard to the nerves, that anything in the form of a thin membrane can perform the functions of a round or thick rope-like substance. Nevertheless, this is seen in the termination of the optic nerve in the retina, and admitted because it is so obvious, for it is so slightly connected with the other membranes as to be easily separated, and examined in the most satisfactory manner. But in some parts of the body, the nerves become interwoven with organs of so firm a texture as not to be capable of the separation necessary for exposing their exact termination; whilst in others this may frequently be seen in the form of a fine membrane. The nerve supplying a muscle terminates in a fine membrane, which is extended among the fibres, and is continuous with that usually termed cellular. When it has resolved itself into this membrane, and has to spread itself over the whole of a muscle, because it cannot be followed into all the fibres, will it be asserted that these are destitute? And if this cannot fairly be maintained, in like manner, when a nerve has been traced to any other part, and its termination found similar to that in the muscle, may it not be concluded that the whole of this part is likewise supplied with nerves?" (P. 34.)

